# Low Phosphatidylserine+ Cells Within the CD34+/CD45dim/CD117(c-kit)+ Subpopulation Are Associated with Poor Outcomes in Metastatic Colorectal Cancer

**DOI:** 10.3390/cancers17030499

**Published:** 2025-02-02

**Authors:** Davide Brocco, Pasquale Simeone, Pietro Di Marino, Domenico De Bellis, Francesca D’Ascanio, Giulia Colasante, Antonino Grassadonia, Michele De Tursi, Rosalba Florio, Mauro Di Ianni, Alessandro Cama, Nicola Tinari, Paola Lanuti

**Affiliations:** 1 Department of Medical, Oral & Biotechnological Sciences, University "G. D’Annunzio", 66100 Chieti, Italy; ntinari@unich.it; 2 Center for Advanced Studies and Technology (CAST), University “G. d’Annunzio”, 66100 Chieti, Italy; pasquale.simeone@unich.it (P.S.); domenico.debellis@unich.it (D.D.B.); francesca.dascanio@unidav.it (F.D.); giulia.colasante001@phd.unich.it (G.C.); paola.lanuti@unich.it (P.L.); 3 Department of Medicine and Aging Sciences, University “G. D’Annunzio”, 66100 Chieti, Italy; 4 Clinical Oncology Unit, S.S. Annunziata Hospital, 66100 Chieti, Italy; 5 Department of Humanities, Law and Economics, “Leonardo da Vinci” University, 66010 Torrevecchia Teatina, Italy; 6 Department of Innovative Technologies in Medicine and Dentistry, University “G. D’Annunzio” 66100 Chieti, Italy; grassa@unich.it (A.G.); detursi@unich.it (M.D.T.); 7 Department of Pharmacy, University “G. D’Annunzio”, 66100 Chieti, Italy; rosalba.florio@unich.it

**Keywords:** colorectal cancer, peripheral biomarkers, circulating endothelial cells, circulating endothelial progenitor cells, circulating pro-angiogenic cells, computational flow cytometry analyses

## Abstract

Background: Colorectal cancer is among the most prevalent causes of tumor-related deaths worldwide. Antiangiogenic therapy represents a cornerstone of metastatic CRC treatment, and biomarkers are advocated for the optimization of this therapeutic strategy. Methods: In this observational prospective study, we employed an optimized flow cytometry protocol to investigate the prognostic and predictive potential of blood circulating endothelial cells (CECs), circulating endothelial progenitor cells (CEPCs), and related subsets in a cohort of patients with metastatic colorectal cancer (*n* = 40). Results: Computational FC analysis revealed a differential enrichment of blood cell clusters with a CD34+/CD45dim/CD117(c-kit)+ phenotype between responders and non-responders both to antiangiogenic and non-antiangiogenic treatments. Intriguingly, our results show that a high percentage of annexin V-negative cells in a putative circulating progenitor population with a CD34+/CD45dim/CD117+ phenotype was correlated with a reduced response to systemic anticancer treatments (*p* = 0.015) and worse overall survival (log-rank *p* = 0.03). In addition, we observed increased blood concentrations of CD34+/CD45dim/CD117+/annexin V- cells in patients with a higher number of metastatic sites (*p* = 0.03). Conclusions: Overall, these findings hold promise for the identification of novel circulating biomarkers to develop more personalized treatment approaches in patients with metastatic colorectal cancer.

## Simple Summary

The aim of this study was to investigate the prognostic and predictive role of blood circulating endothelial cells (CECs), circulating endothelial progenitor cells (CEPCs), and their related subsets in patients with metastatic colorectal cancer treated with antiangiogenic agents. An optimized flow cytometry (FC) protocol was applied to identify and subtype CECs and CEPCs in a cohort of 40 patients affected by metastatic colorectal cancer (mCRC) treated or not treated with antiangiogenic therapy. Our results show that the frequencies of annexin V- cells within the blood CD34+/CD45dim/CD117+ cell subset correlated with the patient cohort’s overall survival and clinical response. These results revealed the promising role of circulating progenitor cells as potential biomarkers in patients with mCRC.

## 1. Introduction

Colorectal cancer (CRC) represents a major global health challenge, since CRC represents the third most common cancer and the second leading cause of cancer mortality worldwide [1,2]. Colorectal tumor progression and metastasis are significantly promoted by neoangiogenesis, which consists in the growth of new blood vessels [3]. Several anti-angiogenic drugs have been developed, and a large number of randomized clinical trials have shown clinical benefit from the employment of this class of antitumoral agents in patients with metastatic colorectal cancer [4,5,6,7]. This has led to the approval of various antiangiogenics for CRC treatment [8]. As a result, antiangiogenic drugs have become pivotal agents in the therapeutic arsenal against metastatic CRC [9]. Despite advances in antiangiogenic therapies, several challenges still need to be addressed, including drug resistance development and limited efficacy in subgroups of patients [10]. Compensatory mechanisms often drive resistance to vascular endothelial growth factor (VEGF) inhibition [11]. Therefore, novel combinations and antiangiogenic drugs are needed [12]. Additionally, more effective predictive biomarkers for antiangiogenic therapies are considered necessary and should be developed to improve patient selection and maximize clinical benefit [13].

In such a context, several cellular subtypes involved in endothelial homeostasis, such as circulating endothelial cells (CECs) and circulating endothelial progenitor cells (CEPCs), have been investigated as potential biomarkers [14,15]. CECs are mature endothelial cells that enter into the bloodstream after detaching from vessel walls because of vascular damage or physiological turnover [16,17,18]. Blood levels of CECs can be hypothetically modulated by vascular remodeling, thus supporting their role as potential circulating reporters of cancer neoangiogenesis and putative biomarkers for antiangiogenic treatment [19]. Conversely, CEPCs are mobilized from the bone marrow and contribute to vascular repair by differentiating into endothelial cells, thus promoting angiogenesis [20,21]. Several reports have highlighted correlations between high blood levels of CEPCs, and more advanced disease in patients with solid tumors have been described [21,22]. In addition, CEPCs are able to cross the blood–brain barrier, thus contributing to tumor vascularization and progression [23]. Of note, higher levels of CEPCs have been correlated with negative clinical outcomes including poor treatment response and worse cancer-related overall survival both in solid tumors and hematological malignancies [24,25,26,27]. CEPCs can also represent targets for anticancer treatments. In this regard, FTY720—an immunomodulatory drug—reduced CEPC levels and suppressed liver tumor metastasis in a rat model, thus suggesting the potential of this drug to prevent tumor recurrence [28]. Some other agents like phloroglucinol have been shown to block tumor angiogenesis by specifically inhibiting CEPC bioactivities [29].

Unfortunately, the identification and quantification of blood CECs and CEPCs is challenging. In particular, there is a phenotypic overlap among CECs, CEPCs and other cell types, hampering the standardization of methods for their detection [30,31]. Flow cytometry is commonly employed to identify CEC and CEPC populations by using specific surface markers.

Therefore, herein, we undertook the task of studying different circulating endothelial subtypes in patients with metastatic colorectal cancer (mCRC) by applying an optimized flow cytometry method based on the use of a large panel of endothelial and progenitor markers. We further used computational flow cytometry methods to automatically identify new putative circulating endothelial subsets related to mCRC outcomes.

## 2. Materials and Methods

### 2.1. Patients

This prospective observational pilot study enrolled adult patients by applying the following inclusion criteria: age ≥ 18 years, histologic or cytologic confirmed diagnosis of stage IV colorectal adenocarcinoma, patients who are candidates for a first or further line of antitumoral systemic treatment for metastatic disease, availability of suitable blood sample for FC analysis, and written informed consent. Patients with an Eastern Cooperative Oncology Group (ECOG) performance status (PS) > 2 or ongoing antitumoral systemic therapy for metastatic disease were considered not eligible for the study. A total of 40 patients were enrolled in the study from the Clinical Oncology Unit of the SS Annunziata Hospital in Chieti (Italy) from January 2017 to August 2022. Any procedures that involve human participants were conducted in accordance with the ethical standards of the 1964 Helsinki Declaration and its later amendments or with comparable ethical standards. This study was approved by the local ethics committee on 25 February 2016.

### 2.2. Peripheral Blood Collection

For each patient, peripheral blood (5 mL) was collected at the baseline by using ethylenediamine tetra-acetic acid (EDTA) tubes (BD Biosciences, San Jose, CA, USA, cat. 368861). Peripheral blood samples were processed within 8 h of blood draw.

### 2.3. Flow Cytometry Assay for the Identification and Count of Circulating Endothelial Cells, Circulating Progenitor Endothelial Cells, and Their Subsets

The identification and count of each detected population was carried out according to a previously published flow cytometry protocol, already optimized and standardized by a network of six laboratories including ours [16,17,18]. Briefly, the proposed panel was established after testing a list of reagents already used in the literature [16,17]. Markers previously shown to be redundant (i.e., CD31) or markers with a debated role in the endothelial lineage identification (i.e., CD133) were excluded from the final panel [18]. We have also paralleled the staining and acquisition protocols to other methods already established [18]. Finally, we have previously standardized the protocol used in the present study and the applied gating strategy by a network study [17]. The panel of markers used for multiparametric flow cytometry analysis is detailed in Table S1.

#### 2.3.1. Blood Processing and Cell Staining

Peripheral blood samples were processed by a common flow cytometry lyse and wash method [16,17,18]. Briefly, for each sample, 5 mL of peripheral blood, harvested in EDTA tubes, as above specified (Section 2.2), underwent an erythrocyte lysis step being treated with 45 mL of Pharm Lyse solution (BD Biosciences, San Jose, CA, USA) for 15 min at room temperature under gentle agitation. Samples were then centrifuged at 400 g for 10 min at room temperature and washed by adding 2 mL of PBS. The pellet was resuspended with 100 µL of 1X binding buffer (BD Biosciences, San Jose, CA, USA), and the surface staining was carried out by adding the mixture of reagents summarized in Table S1. Samples were then incubated for 30 min at 4 °C and washed with 2 mL of 1X binding buffer. Before the acquisition, samples were re-suspended in 1.5 mL of 1X binding buffer (BD Biosciences) and filtered using 70 μm filters. Finally, 10 × 10^6^ events per sample were acquired by flow cytometry (BD FACSCanto II, BD Biosciences, San Jose, CA, USA).

#### 2.3.2. Flow Cytometry Computational Analysis

An unsupervised computational analysis of flow cytometry data was carried out by applying the t-distributed Stochastic Neighbor Embedding (t-SNE) and FlowSom algorithms. To this end, plugins from FlowJo software (BD Bioscience, San Jose, CA, USA) v 10.10.0 were employed. Flow cytometry data—derived from patient samples within the same study group—were merged into a single file. T-SNE was run on concatenated data with a perplexity parameter of 30 and 1000 iterations. The FlowJo plugin FlowSOM (v.4.1.0) was applied to concatenated data by setting a metacluster number of 6 and an SOM grid size of 10 × 10. Compensated parameters were used for both t-SNE and FlowSom calculations.

#### 2.3.3. Identification and Enumeration of Cell Subsets by Manual Gating

Data were analyzed using FACSDiva v 6.1.3 (BD Biosciences, San Jose, CA, USA) and FlowJo v 10.10.0 (BD Bioscience, San Jose, CA, USA), utilizing a dual platform counting method and using the lymphocyte subset as the reference population and applying the following formula [16,17,18]:

Abs Population of Interest/mL = (Population of Interest Abs Count*(Lymphocyte Count)/mL)/(# Lymphocyte count) where Abs: absolute; Abs Population of Interest/mL: Concentration.

The possibility of parallelling the results all along the whole study was ensured by the daily Cytometer Setup and Tracking (CS&T) Beads, used both to initially generate the instrument (setting target values) and to ensure the proper performance of the instrument.

### 2.4. Statistical Analysis

Statistical analysis was performed using Graphpad Prism 9 (GraphPad Software Inc.; San Diego, CA, USA) and SPSS v25.0 (IBM SPSS, Chicago, IL, USA). The normality of the data was assessed using the Shapiro–Wilk test. Comparisons were made by applying the unpaired *t*-test for normally distributed data, whereas the Mann–Whitney and Kruskal–Wallis non-parametric tests were used for non-normally distributed data. Multiple comparisons were assessed using Dunn’s test. Correlations between blood levels of the rare cell subpopulation and clinical–pathological variables were assessed by using Spearman’s rank correlation coefficients. A proportion of clinical variables including ECOG PS, number and site of metastasis, diabetes, cardiovascular disease, arterial hypertension, body mass index (BMI), tumor grading, serum blood CEA concentration, tumor location, and mutational status of the K-RAS gene were collected retrospectively and included in the correlation analysis. Radiological response was evaluated according to RECIST criteria v1.1. The overall response rate (ORR), calculated as the percentage of patients achieving complete response (CR) or partial response (PR), was calculated to discriminate between responders and non-responders. Overall response rates were compared between patient groups by applying Fisher’s exact test. Receiving operative curves (ROCs) of response vs. non-response were calculated to evaluate the predictive abilities of selected cell subsets. The Youden Index was employed to calculate the optimal cut-off points using ROC curve data. Univariate and multivariate Cox proportional hazards models were used to calculate the hazard ratios (HRs) with 95% of confidence intervals (CIs). Internal validation was carried out with the SPSS biased–corrected and accelerated bootstrap method with 1000 bootstrap samples and a 95% confidence interval. The Kaplan–Meier (KM) curve estimator was applied to estimate median overall survival (mOS), and the log-rank test was employed to examine differences in mOS across patient groups. A *p*-value of <0.05 was considered statistically significant.

## 3. Results

### 3.1. Machine Learning Algorithms Reveal Specific Subsets of Cells with a CD34+/CD45-/dim Phenotype in mCRC Responders vs. Non-Responders to Antitumoral Systemic Therapies

**Figure 1:**
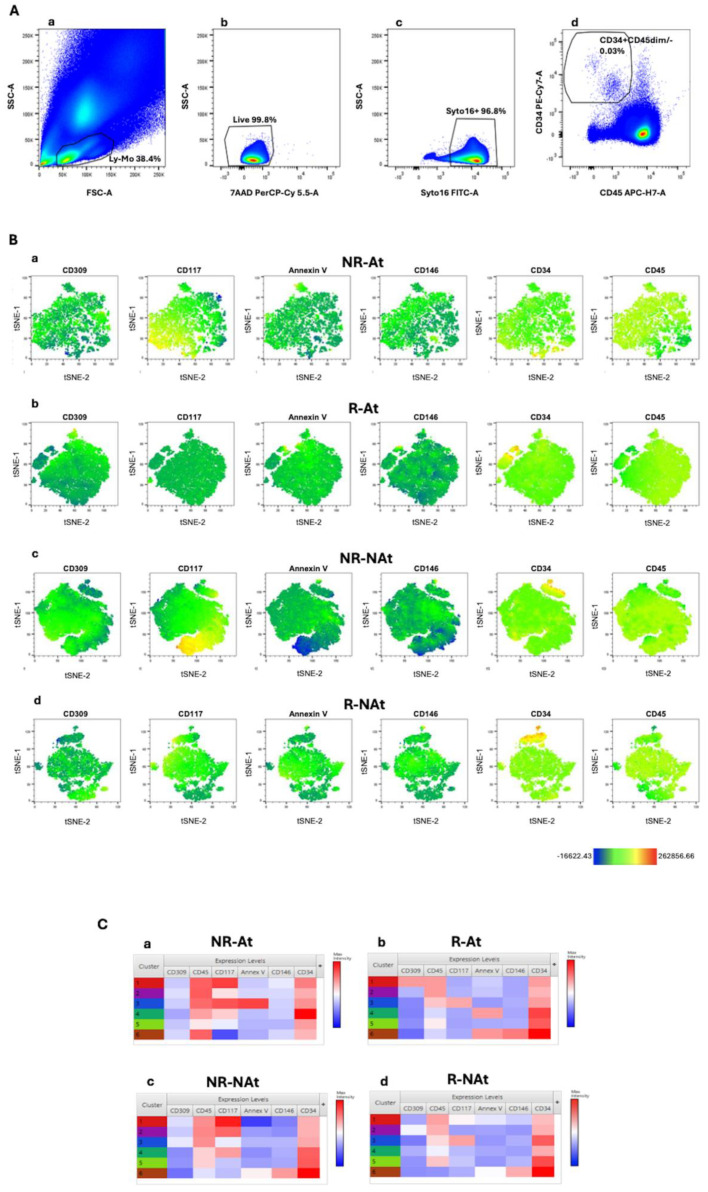
Flow cytometry computational analysis of blood cells with CD34+ CD45-/dim phenotype. (**A**) Gating strategy for the identification of blood circulating CD34+CD45-/dim cells. (**a**) Lympho-monocytes (Ly-Mo) were firstly selected on a forward scatter area (FSC-A)/side scatter-A (SSC-A) pseudo-color plot. (**b**) Alive and (**c**) nucleated cells were identified according to 7-aminoactinomycin D (7-AAD) negativity and positivity to the nuclear vital marker Syto16, respectively. (**d**) Alive and nucleated lympho-monocytes were analyzed on a CD34/CD45 plot, and CD34+CD45dim/- were selected. (**B**) T-SNE dot plots showing distribution of single marker expression in the following groups: (**a**) non-responders to antiangiogenic therapies (NR-At), (**b**) responders to antiangiogenic therapies (R-At), (**c**) responders to non-antiangiogenic therapy (R-NAt) and (**d**) non-responders to non-antiangiogenic therapy (NR-NAt). (**C**) Heatmaps reporting phenotypical features of cell clusters calculated by FlowSOM analysis in the same study groups (**a**–**d**). Data are representative of all reported patients.

We carried out an exploratory computational analysis to explore flow cytometry data generated using a multiparametric panel, as described in the method section. This panel was applied to identify blood circulating endothelial cells, progenitor cells, and related subsets, as reported. In line with the power of analysis of this machine learning approach, this exploratory analysis was carried out in four groups of three mCRC patients—responders to antiangiogenic-based therapy (R-At), non-responders to antiangiogenic therapy (NR-At), responders to non-antiangiogenic therapy (R-NAt), and patients unresponsive to non-antiangiogenic therapy (NR-NAt). All patients were candidates for first-line antitumoral systemic treatment. Flow cytometry data from single patients were merged within each group before computational analysis. This approach aimed to automatically identify clinically relevant cell clusters for further analyses, while avoiding noise and overfitting that could be generated in large heterogeneous cohorts. Endothelial and pro-angiogenic cell subsets lack expression of or only dimly express the hematopoietic cell marker CD45 [32,33], while expressing CD34 [34]. We applied t-SNE to analyze flow cytometry data from the whole CD34+CD45-/dim blood cell population, while reducing data dimensionality to visualize cell clusters. The gating strategy used to identify CD34+CD45-/dim cells is depicted in Figure 1A T-SNE was run with the following parameters: CD45, CD34, CD146, CD309, Annexin V, and CD117. These markers were selected given that they have been associated with mature (CD146) or progenitor (CD309 and CD117) endothelial cell phenotypes [18,35,36]. Results from t-SNE analysis are represented in Figure 1. Globally, t-SNE plots showed a separation between the subset of cells with the CEC phenotype (CD34 bright expression) and cell clusters with lower CD34 surface expression. Of note, the analysis of t-SNE plots by single markers revealed that CD117-expressing cells were more represented in non-responders from both treatment groups, as compared to responders (Figure 1B). We further analyzed flow cytometry data using FlowSOM to carry out hierarchical clustering and improve the identification of distinct cell subsets. FlowSOM was run with the same parameters selected for t-SNE analysis. Figure 1C shows heatmaps depicting the phenotypic features of cell clusters derived from the application of the FlowSOM algorithm to flow cytometry data in each patient group. Notably, confirming t-SNE results, cell clusters with the CD34+/CD45dim phenotype and expressing CD117 were predominant in non-responders, as compared to responders. The subset characterized by the CD34+/CD45dim/CD117+ phenotype presented heterogeneity for phosphatidylserine surface expression (revealed by annexin V) in non-responders, whereas these cell subsets did not appear to co-express CD146 and CD309 (VEGFR-2) both in responders and non-responders. Additionally, a cluster of VEGFR-2-expressing CD34+/CD45dim cells was detectable in the group of responders to antiangiogenic agents (Figure 1C). The subset of cells with high CD34 expression and negative for CD45—referred to as CEC phenotype—was equally represented across all patient groups. Overall, this exploratory analysis with machine learning algorithms suggested that blood CD34+/CD45dim/CD117+ cells might be associated with tumor resistance to both antiangiogenic and non-antiangiogenic therapies. Of note, heterogeneity in phosphatidylserine expression was observed among cell clusters with a CD34+/CD45dim/CD117+ phenotype.

### 3.2. Blood Levels of Peripheral Blood Cells with a CD34+/CD45dim/CD117+/AnnV- Phenotype Are Correlated with Overall Response Rate to Antitumoral Systemic Therapies in Patients with mCRC

To validate the results obtained by exploratory flow cytometry computational analyses, we investigated the relationship between blood concentrations of cells with CD34+/CD45dim/CD117+ phenotype and response to antitumoral systemic therapy in the whole cohort of enrolled mCRC patients (*n* = 40). Overall baseline demographic and clinical–pathological characteristics of patients included in the study are reported in Table 1.

**Figure 2:**
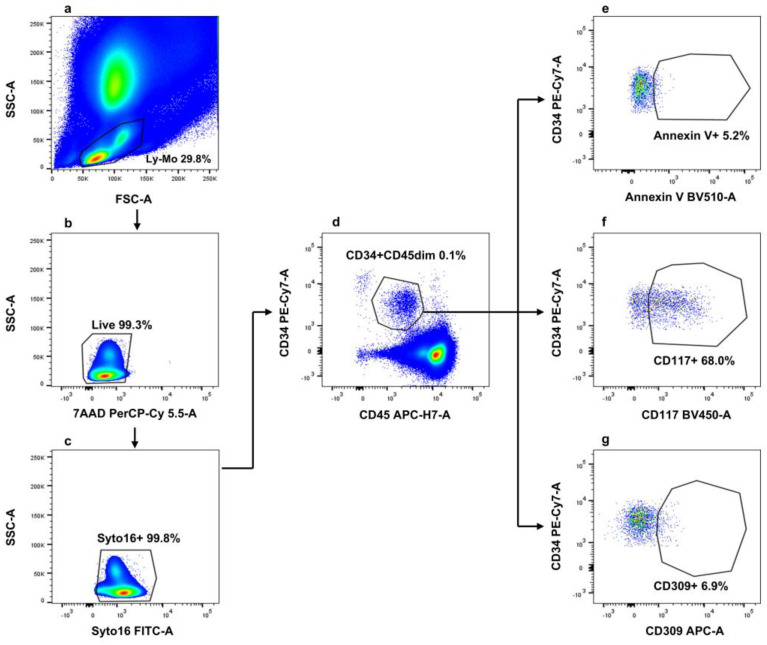
Flow cytometry gating strategy for the identification of circulating endothelial, endothelial progenitor, and pro-angiogenic cells. (**a**) Lympho-monocytes (Ly-Mo) were firstly selected on a forward scatter area (FSC-A)/side scatter-A (SSC-A) pseudo-color plot. (**b**) Dead cells were further excluded on the basis of their positivity to 7-aminoactinomycin D (7-AAD), and (**c**) nucleated circulating cells were identified based on their positivity to the nuclear vital marker Syto16. (**d**) Circulating events displaying lympho-monocyte scatter properties, both alive and nucleated, were analyzed for their CD34/CD45 expression, and as evidenced, two populations expressing different levels of CD34 were identified; these were a larger population of cells expressing CD34 and dim levels of CD45 (CD34+/CD45dim) and a smaller subset exposing higher levels of CD34 and being negative for CD45 (CD34bright/CD45-). Cells with a CD34+/CD45dim phenotype were analyzed for (**e**) phosphatidylserine exposure (revealed by annexin V), (**f**) CD117 (also known as c-kit), and (**g**) CD309 (which is VEGFR-2). This gating strategy was applied to all analyzed samples (*n* = 40).

Blood CD34+/CD45dim/CD117+cells were identified and enumerated by conventional polychromatic flow cytometry, as reported. In line with findings from hierarchical clustering analysis, annexin V-positive and -negative events were also evaluated by manual gating. Results underlined that two distinct cell subsets—CD34+/CD45dim/CD117+/AnnV-and CD34+/CD45dim/CD117+/AnnV+—composed the CD34+/CD45dim/CD117+ circulating cell population. The used flow cytometry gating strategy is depicted in Figure 2.

**Figure 3:**
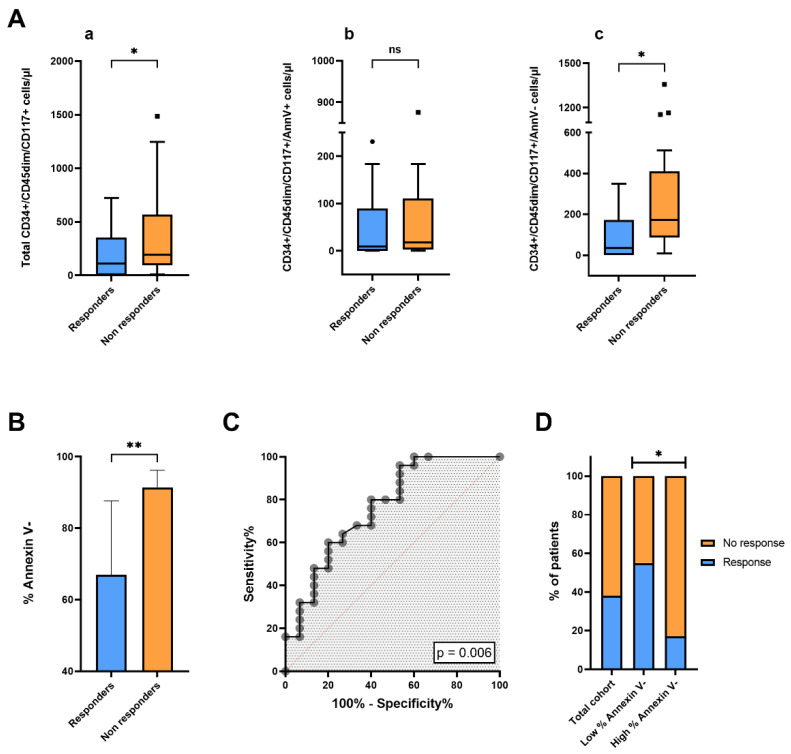
Correlation between response to systemic anticancer agents and concentrations and frequencies of CD34+CD45dimCD117+ cells and their subsets. (**A**) Box plots showing differences in blood concentrations of (**a**) total CD34+/CD45dim/CD117+ cells, (**b**) CD34+/CD45dim/CD117+/Ann V+ cells, and (**c**) CD34+/CD45dim/CD117+/Ann V- cells between responders and non-responders. Circles represent outliers. (**B**) Bar charts illustrating comparison of percentages of annexin V-negative events within CD34+/CD45dim/CD117+ cells between responders and non-responders. Statistical comparisons were performed by *t*-test or Mann–Whitney U test. (**C**) ROC curve showing the effect of percentage of annexin V- cells with a CD34+/CD45dim/CD117+ phenotype in predicting treatment response. (**D**) Bar charts depicting patient distributions according to radiological response in the total cohort and in the two subgroups of patients with low and high % of annexin V-negativity within the CD34+/CD45dim/CD117+ cell population. Statistical comparison was carried out by Fisher’s exact test. *, *p* <0.05; **, *p* < 0.01.

Patients were separated into two subgroups according to overall response rate (ORR). Partial or complete response after antitumoral systemic therapy was achieved in 15 of 40 patients (ORR = 37.5%), while progressive or stable disease was observed in 25 of 40 patients. We compared blood concentrations of total CD34+/CD45dim/CD117+, CD34+/CD45dim/CD117+/AnnV+ and CD34+/CD45dim/CD117+/AnnV- cells at baseline between responders (*n* = 15) and non-responders (*n* = 25) (Figure 3A(a–c)). Blood levels of total CD34+/CD45dim/CD117+ cells were significantly lower in responders, as compared to non-responders (*p* = 0.03). Interestingly, the difference in blood concentrations of CD34+/CD45dim/CD117+cells between responders and non-responders was mainly driven by the different concentrations of CD34+/CD45dim/CD117+/AnnV- cells between the two groups of patients (*p* = 0.01). Indeed, no significant difference in blood concentrations of CD34+/CD45dim/CD117+/AnnV+ cells was observed between responders and non-responders (*p* = 0.58). Accordingly, we observed that the median percentage of CD34+/CD45dim/CD117+ cells negative for annexin V at baseline was significantly lower in patients with tumor response, as compared to those with stable or progressive disease (*p* = 0.005) (Figure 3B). As depicted in Figure 3C, the ROC curve analysis provided evidence of a correlation between treatment response and blood percentage of annexin V- cells within the CD34+/CD45dim/CD117+ subset (AUC = 0.764 [CI 95% 0.607–0.921]; *p* = 0.006). By applying the Youden Index to ROC curve data, we calculated the optimal cut-off to dichotomize the population in patients with high and low percentages of annexin V- cells (cut-off = 90%); we further compared overall response rates between the two groups (Figure 3D). Of note, patients in the group with higher percentages of Annexin V- cells presented a 4-fold lower ORR, as compared with patients with lower % of Annexin V- events within blood circulating CD34+/CD45dim/CD117+ cells (ORR% 16.7 vs. 54.5; *p* = 0.015).

Furthermore, machine learning analysis suggested a potential predictive role of CD34+/CD45dim/CD309(VEGFR-2)+ cells in patients treated with antiangiogenic-based therapies. Therefore, we analyzed the blood concentrations of this subset of blood-derived VEGFR2+-expressing cells at baseline in the subgroup of mCRC patients who received antiangiogenic agents (*n* = 15). We did not observe, however, any difference in blood concentration of VEGFR-2+ cells between responders and non-responders to antiangiogenic drugs (*p* = 0.27) (Supplementary Figure S1).

### 3.3. Blood-Circulating Concentration of CD34+/CD45dim/CD117+/Annexin V- Cells Correlates with the Number of Metastatic Sites

**Figure 4:**
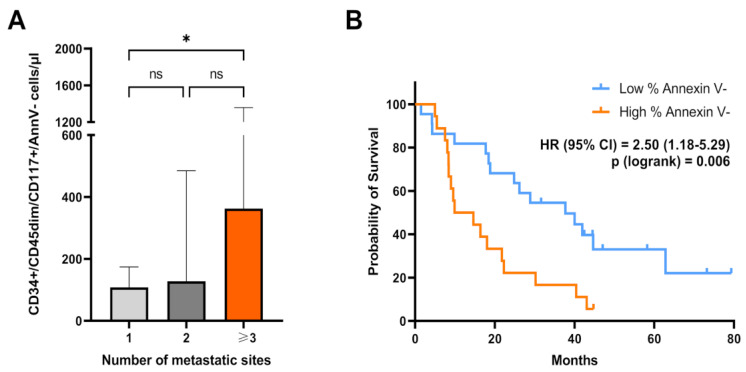
(**A**) Bar charts showing differences in blood concentrations of CD34+/CD45dim/CD117+/Ann V- according to the number of metastatic sites in the total patient cohort. Statistical comparisons were performed using the Kruskal–Wallis test with adjustment for multiple comparisons. (**B)** Kaplan–Meier (KM) curves depicting the relationship between overall survival and the percentage of annexin V-negative cells with a CD34+/CD45dim/CD117+ phenotype. The log-rank test was used for statistical comparisons. One asterisk (*) indicates *p* value smaller than 0.05 (*p* < 0.05).

We evaluated the correlation between clinical–pathological factors and blood levels of circulating CD34+/CD45dim/CD117+, as well as CD34+/CD45dim/CD117+/AnnV- cells. The correlation analysis included the following clinical–pathological variables: sex, ECOG PS, age, number of metastatic sites, tumor grading, lung metastasis, liver metastasis, body mass index, primary tumor location, K-RAS mutational status, serum blood CEA concentration, and number of previous lines of systemic therapies in the overall patient cohort (Table S2). Notably, the blood concentration of CD34+/CD45dim/CD117+/AnnV- cells was correlated with the number of metastatic sites (*p* = 0.03). The median blood concentration of cells with the CD34+/CD45dim/CD117+/AnnV- phenotype was almost 2-fold higher in patients with multiple organ involvement (>3 site of metastasis), as compared with those with single-site metastasis (*p* = 0.03) (Figure 4A). Additionally, blood concentrations of total CD34+/CD45dim/CD117+ cells were significantly and positively correlated with lung metastatic spread (*p* = 0.04) (Supplementary Table S2). There was also a weaker trend for a positive correlation between CD34+/CD45dim/CD117+/AnnV- and lung metastasis, but it did not reach statistical significance (*p* = 0.06). No other significant correlations were observed (Supplementary Table S2).

### 3.4. A High Percentage of Blood Annexin V- Cells with a CD34+/CD45dim/CD117+ Phenotype Independently Predicts Worse Survival in Patients with mCRC

Considering the association observed between CD117+ cell subsets and tumor response, we investigated whether baseline blood concentrations of CD117-expressing CD34+/CD45dim cells and the percentage of annexin V-negative cells within this cell subset were associated with survival in patients with mCRC (*n* = 40). Univariate and multivariate Cox proportional hazards regression analyses were employed to investigate the correlation between patient survival and cell subsets. On univariate analysis, a significant correlation between overall survival and the percentage of annexin V- blood cells with a CD34+/CD45dim/CD117+ phenotype was observed (*p* = 0.04) (Table 2). No correlation between blood concentration of the whole CD34+/CD45dim/CD117+ cell population and survival was found (*p* = 0.23). Univariate Cox proportional hazards regression analysis was also employed to explore the correlation between OS and clinical–pathological variables including ECOG PS, age, number of metastatic sites, BMI, tumor grading, primary tumor location, serum blood CEA concentration, K-RAS mutational status, and line and type of systemic therapy (Table 2). In this regard, ECOG PS and CEA levels correlated with survival (*p* = 0.001; *p* = 0.03, respectively). Of note, no correlation between the number of metastases and overall survival was observed (*p* = 0.91; *p* = 0.98). Cox regression univariate analyses were verified via bootstrap validation. All variables significantly correlated with OS (*p* < 0.05) in the univariate analysis, and those considered clinically meaningful—including line and type of systemic therapy received after study enrollment—were selected as candidate variables for the multivariate analysis. A Cox proportional hazards regression multivariate analysis employing a stepwise backward procedure was used to obtain a final model of the variables that are independently correlated with survival. In this model, a variable was excluded stepwise if the corresponding *p* value was >0.10. Intriguingly, in the final multivariate model, the percentage of annexin V expression within blood CD34+/CD45dim/CD117+ cells was found to be independently associated with survival in our cohort of mCRC (Table 2).

The difference in overall survival between groups with a high and low percentage of annexin V- cells (cut-off = 90%) is depicted in the Kaplan–Meier plot reported in Figure 4. Kaplan–Meier (KM) survival curves indicated that patients with a higher percentage of annexin V- cells in the circulating CD34+/CD45dim/CD117+ cell compartment presented a remarkably reduced survival, as compared to patients with a lower percentage of annexin V- events (*p* = 0.006) (Figure 4B). No difference was observed between patients with different blood levels of total CD117-expressing CD34+/CD45dim cells (cut-off= 135 cells/µL; (*p* = 0.24) (Supplementary Figure S2).

## 4. Discussion

Tumour growth is sustained by the formation of new blood vessels in a process called neoangiogenesis. Targeting neoangiogenesis has represented a challenge within cancer therapy in recent decades [37]. Several antiangiogenic drugs have recently been developed, such as monoclonal antibodies or tyrosine kinase inhibitors [38]. They play crucial roles in the treatment of colorectal cancer by inhibiting the formation of new blood vessels necessary for tumor growth and metastasis. These drugs target the vascular endothelial growth factor (VEGF) pathway, which is of pivotal importance for neoangiogenesis in CRC and other solid tumors [39]. Despite progress in antiangiogenic therapies for advanced CRC, there are still unmet challenges to address, including drug resistance and the limited efficacy of this treatment strategy in patient subgroups. Therefore, the assessment of more effective predictive biomarkers for antiangiogenic and, more widely, antitumoral systemic therapy is a clinical need of growing interest [13]. Several cellular subtypes involved in endothelial homeostasis, such as circulating endothelial cells (CECs), circulating endothelial progenitor cells (CEPCs), and pro-angiogenic hematopoietic stem cells (HSCs), have potential as biomarkers in this context. Therefore, we undertook the task of deeply analyzing and correlating blood levels of circulating endothelial cells and their putative progenitor cells with clinical outcomes in mCRC patients.

In this study, we used computational flow cytometry analysis for identifying novel cell subsets of clinical relevance. This approach allows for automatic detection of cell populations and extraction of meaningful biological information from high-dimensional datasets [40,41]. Interestingly, we applied such a method to a large flow cytometry panel that included markers of putative CECs and CEPCs. It is known that endothelial and pro-angiogenic cell subsets lack the expression of or only dimly express hematopoietic cell marker CD45 [32,33]. Conversely, endothelial cells, endothelial progenitors, and pro-angiogenic circulating cells express CD34 [34]. Interestingly, by applying automatic data analysis to the circulating CD34+CD45dim/neg cell population, we observed—in an unbiased fashion—distinct distributions in cell subsets between mCRC responders and non-responders to antitumoral systemic therapies. Thus, in silico analysis provided specific flow cytometry signatures related to tumor response that would hardly be obtained with classical analysis of bidimensional data. In detail, the flow cytometry computational analysis of circulating CD34+CD45dim/- cells showed differential expression of CD117+ cell clusters between responders and non-responders. In line with these findings, conventional flow cytometry data analysis of blood concentrations of circulating CD34+/CD45dim subsets in a cohort of 40 patients with mCRC confirmed that non-responders displayed higher circulating levels of CD117-expressing cells, as compared with responders. This phenotype may correspond to cells with endothelial progenitor features [42,43,44,45].

Of note, we observed that a subset of the CD34+/CD45dim/CD117+ parental population not featuring phosphatidylserine (AnnV-) had a high capability to predict treatment efficacy. More in detail, non-responders displayed higher concentrations of circulating CD34+/CD45dim/CD117+/AnnV- than responder patients. Interestingly, phosphatidylserine (PS) exposure on endothelial cells can be induced by different stimuli, such as oxidative stress and inflammatory cytokines [46]. PS is externalized on the vascular endothelium in different tumor models, and this externalization is driven by tumor-associated oxidative stress and activating cytokines [47]. On the other hand, PS is externalized from the inner leaflet to the outer leaflet of the plasma membrane, acting as an "eat-me" signal to direct phagocytes to engulf PS expressing cells [48,49]. It is also known that stem cell factor (SCF), the ligand of CD117, protects tumor cells from apoptosis via an autocrine loop [50]. Thus, annexin V+ cells may represent cellular elements undergoing apoptosis or detaching from vessel walls. Conversely, cells with a CD34+/CD45dim/CD117+/AnnV- phenotype may make up an active proliferating subpopulation of circulating progenitors with a potential role in tumor progression [32]. It has been shown that CD117+CEPCs differentiate into endothelial cells and form new blood vessels within tumors, supporting tumor growth and metastasis [51,52]. The recruitment of these cells is often mediated by tumor-derived factors, such as VEGF and stem cell factor (SCF), that attract and stimulate the differentiation of CD117+CEPCs [53]. This process is crucial for the formation of a functional tumor vasculature that supports cancer progression. It is also known that chemotherapy and growth factors, like granulocyte colony-stimulating factor (G-CSF), induce the mobilization of different stem and progenitor cell subtypes, including CD117+CEPCs, from the bone marrow to peripheral blood [54,55]. This mobilization is essential for the formation of new blood vessels that, in turn, support tumor growth and metastasis [55]. Conversely, targeted therapies such as motesanib, an inhibitor of VEGF and Kit receptors, have been shown to reduce CD117+CEPC levels, correlating with antitumor activity [56]. These data were corroborated by the observation that high frequencies of annexin V- cells within the circulating CD34+/CD45dim/CD117+ population were independently associated with worse survival in our cohort of mCRC patients. Patients displaying a population of CD34+/CD45dim/CD117+ circulating cells, almost totally composed of annexin V-negative events (>90%), harbored a more aggressive disease. This may be due to the potential role of annexin V-/CD117+ cells that could be recruited from the bloodstream to the tumor, where they may become active participants in tumorigenesis [57]. This hypothesis is sustained by a large body of literature showing that the expression of c-Kit (CD117) within solid tumors is associated with cancer stemness, treatment resistance, tumor progression, and metastasis [58,59,60].

Additionally, we observed that concentrations of the same CD34+/CD45dim/CD117+/AnnV- cell subpopulation in peripheral blood were correlated with the number of metastatic sites. Specifically, the median blood cell concentration of CD34+/CD45dim/CD117+/AnnV- cells was almost 2-fold higher in patients with multiple organ involvement (≥3 sites of metastasis), as compared to those with single-site metastasis. Expression of CD117 on cells of the tumor microenvironment (TME) may influence metastatic tumor spread through various mechanisms. In a mouse model of breast cancer associated with arthritis, the interaction between mast cell CD117+ and stem cell factor (SCF) released by tumor cells enhanced metastasis by remodeling both the TME and the metastatic niche [61]. Furthermore, CD117+ adipose tissue-derived mesenchymal stem cells promote breast cancer growth and angiogenesis, further supporting the role of CD117 in metastasis [62]. Conversely, it is conceivable that expansion of the blood CD34+/CD45dim/CD117+/AnnV- cell compartment may be secondary to increased tumor burden, which may perturbate blood levels of this cell subset [57].

## 5. Conclusions

Altogether, our data suggest a role for blood CD34+/CD45dim/CD117+/AnnV- cells in mCRC treatment resistance and progression. Therefore, blood circulating CD34+/CD45dim/CD117+/AnnV- cells may represent a candidate biomarker for predicting clinical outcomes in patients with mCRC. This intriguing observation calls for further analysis in larger cohorts in order to gain a deeper understanding of the pathological significance of this cell subpopulation and its potential as a biomarker in colorectal cancer.

## Figures and Tables

**Table 1:** Demographic and clinical characteristics of enrolled mCRC patients (*n* = 40).

Variable	
**Sex (%)** Male Female	29 (72.5) 11 (27.5)
**Median Age (IQR)**	69 (17.8)
**ECOG PS (%)** 0 1	21 (52.5) 19 (47.5)
**Median BMI (IQR)**	26.5 (8.5)
**Median serum CEA ng/mL (IQR)**	14.8 (133.7)
**Diabetes (%)** Yes No Missing	2 (5.0) 35 (87.5) 3 (7.5)
**Hypertension** Yes No Missing	21 (51.5) 17 (42.5) 2 (5.0)
**Cardiovascular disease** Yes No Missing	17 (42.5) 16 (40.0) 7 (17.5)
**Tumor location** Rectum Right Colon Left Colon	15 (37.5) 8 (20.0) 17 (42.5)
**K-RAS mutational status** Wild-type Mutated	22 (55.0) 18 (45.0)
**Tumor grading** G1-2 G3	35 (87.5) 5 (12.5)
**Liver metastasis** Yes No	32 (80.0) 8 (20.0)
**Lung metastasis** Yes No	12 (70.0) 28 (30.0)
**Number of metastatic sites (%)** 1 2 ≥3	23 (57.5) 11 (27.5) 6 (15.0)
**Line of therapy** First-line Second/third-line	31 (77.5) 9 (22.5)
**Systemic Therapy** Chemotherapy + Cetuximab/Panitumumab Chemotherapy + Bevacizumab Chemotherapy + Aflibercept Chemotherapy Regorafenib Cetuximab/Panitumumab Lonsurf	14 (35.0) 14 (35.0) 2 (5.0) 7 (17.5) 1 (2.5) 1 (2.5) 1 (2.5)

**Table 2:** Univariate and multivariate Cox proportional hazards model predicting OS in a cohort of patients with metastatic colorectal cancer (*n* = 40).

	Univariate Analysis		Bootstrap Results (1000 Replicas)				Multivariate Analysis	
Variable	HR (95% CI)	*p*.	Bias	SE	95% CI	*p*.	HR (95% CI)	*p*.
**Total** **CD34+/CD45dim/Cd117+ cells/µL**								
Continuous variable	1.00 (1.00–1.00)	0.23	0.00	0.00	−0.00 to 0.00	0.45		
**% Annexin-** **CD34+/CD45dim/Cd117+ cells**								
Continuous variable	1.01 (1.00–1.03)	0.04	0.001	0.01	−0.00 to 0.21	0.01	1.01 (1.00–1.02)	0.03
**ECOG PS** 0	1 [reference]							
1	3.94 (1.75–8.87)	0.001	0.21	0.42	0.64 to 2.28	0.001	4.10 (1.77–9.31)	0.001
**Age (years)**								
Continuous variable	1.01 (0.98–1.05)	0.53	0.00	0.02	−0.02 to 0.05	0.48		
**Body Mass Index**								
Continuous variable	1.02 (0.95–1.10)	0.62	-0.01	0.04	−0.07 to 0.08	0.57		
**CEA**								
Continuous variable	1.00 (1.00–1.00)	0.03	-0.00	0.00	−0.00 to 0.00	0.004		
**Tumor grading** 1–2	1 [reference]							
3	2.67 (0.98–7.26)	0.05	0.56	0.45	0.29 to 2.06	0.01 ^a^		
**Primary tumor location** Right Colon Left Colon	1 [reference] 0.58 (0.23–1.45)	0.24	−0.09	0.87	−2.20 to 0.58	0.33		
Rectum	0.44 (0.17–1.14)	0.43	−0.12	0.88	−2.45 to 0.20	0.15		
**K-RAS mutational status** Mutated	1 (reference)							
Wild-type	0.97 (0.49–1.95)	0.94	0.01	0.36	−0.74 to 0.69	0.94		
**Number of metastatic sites** 1 2	1 [reference] 1.05 (0.39–2.87)	0.91	−0.03	0.69	−1.15 to 1.32	0.91		
3	0.98 (0.32–2.94)	0.98	−0.01	0.75	−1.37 to 1.35	0.98		
**Line of therapy** First-line	1 [reference]							
Second/third-line	1.02 (0.97–4.75)	0.06	0.01	0.36	−0.16 to 1.57	0.02		
**Systemic therapy** Antiangiogenic therapy No antiangiogenic therapy	1 [reference] 0.55 (0.27–1.11)	0.09	−0.04	0.38	−1.39 to 0.06	0.09	0.48 (0.23–0.99)	0.047

^a^ based on 997 samples; Abbreviations: HR: hazard ratio; SE: standard error; CI: confidence interval

## Data Availability

The data that support the findings of this study are available from the corresponding author, D.B. (Davide Brocco), upon reasonable request.

## Supplementary Materials

The following supporting information can be downloaded at: https://www.mdpi.com/article/10.3390/cancers17030499/s1, Table S1: List of flow cytometry specificities and reagents for cellular analysis of circulating endothelial cells, hematopoietic stem cells, and their subtypes; Figure S1: Blood levels of CD34+/CD45dim/CD309+ cells in responders and non-responders to antiangiogenic treatments; Table S2: Spearman rank correlation coefficients between blood CD34+CD45dim cell subsets and selected clinical–pathological features in patients with mCRC (*n* = 40); Figure S2: Overall survival according to blood levels of CD34+/CD45dim/CD117+ cells.
