# Supplementary material for: Low Phosphatidylserine+ Cells Within the CD34+/CD45dim/CD117(c-kit)+ Subpopulation Are Associated with Poor Outcomes in Metastatic Colorectal Cancer
Source: Cancers (Basel). 2025 Feb 2;17(3):499. doi: 10.3390/cancers17030499 (PMC11816280; doi:10.3390/cancers17030499)
Supplement: Supplementary file 1 [file cancers-17-00499-s001.zip › cancers-3425182-supplementary.pdf]

Supplementary Table 1. List of flow cytometry specificities and reagents for cellular analysis of circulating endothelial cells, circulating endothelial progenitors and their subsets.

Reagent	Fluorochrome	Vendor	Clone	Cat. Number	Volume/ test (µl)
7-AAD	-	BD Bioscences	-	555815	10
Syto16	-	Thermo Fisher Scientific	-	S-7578	1uM
CD34	PE-Cy7	BD Bioscences	8G12	348811	10
CD45	APC-H7	BD Bioscences	2D1	560178	10
CD117	BV421	BD Bioscences	YB5.B8	562434	5
Annexin V	BV510	BD Bioscences	-	561501	5
CD309	APC	BD Bioscences	89106	560495	20
CD146	PE	BD Bioscences	P1H12	550315	40
7-AAD: 7-Aminoactinomycin D; PE					

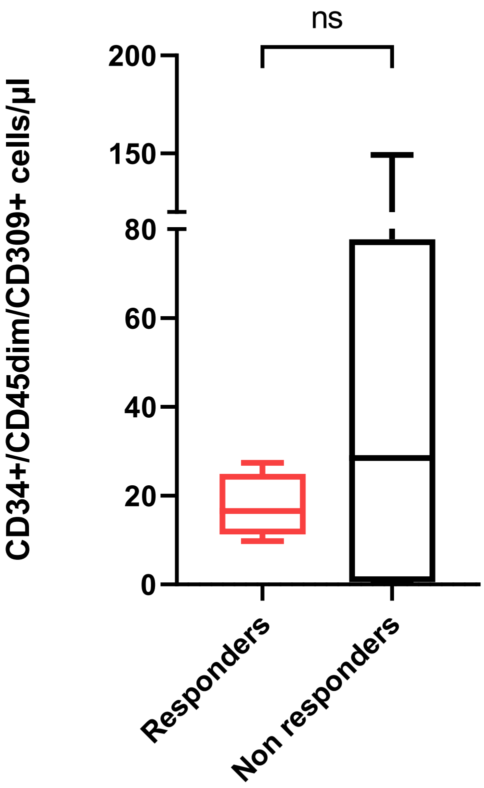

Supplementary Figure 1. **Blood levels of CD34+/CD45dim/CD309+ cells in responders and non-responders to antiangiogenic treatments**. Box plots illustrating median blood concentration of CD34+CD45dimCD309+ cells in responder and non-responders to antiangiogenic-based therapies. Statistical comparison was performed using the Mann-Whitney U test.

Supplementary Table 2. Spearman rank correlation coefficients between blood CD34+CD45dim cell subsets and selected clinical-pathological features in patients with mCRC (n=40)

	**CD34+/CD45dim/CD117+ cells/ul**		**CD34+/CD45dim/CD117+/AnnV- cells/ul**	
	Correlation coefficient	Sig. (2-tailed)	Correlation coefficient	Sig. (2-tailed)
**Sex**	0.172	0.29	0.095	0.56
**ECOG PS**	0.023	0.89	-0.093	0.57
**Age**	0.035	0.83	-0.095	0.56
**BMI**	-0.029	0.86	0.043	0.79
**Diabetes**	0.157	0.35	0.134	0.43
**Arterial Hypertension**	-0.022	0.90	0.043	0.80
**CVD**	-0.045	0.81	0.022	0.90
**Primary tumour location**	-0.075	0.64	-0.118	0.47
**Tumour grading**	0.221	0.18	0.131	0.42
**KRAS mutational status**	0.000	1.00	0.035	0.83
**Liver metastasis**	-0.108	0.51	-0.027	0.87
**Lung metastasis**	**0.321***	**0.04**	0.298	0.06
**CEA**	-0.169	0.30	-0.193	0.23
**Previous line of therapy**	0.064	0.70	0.075	0.65
**Number of metastatic sites**	0.270	0.09	**0.337***	**0.03**

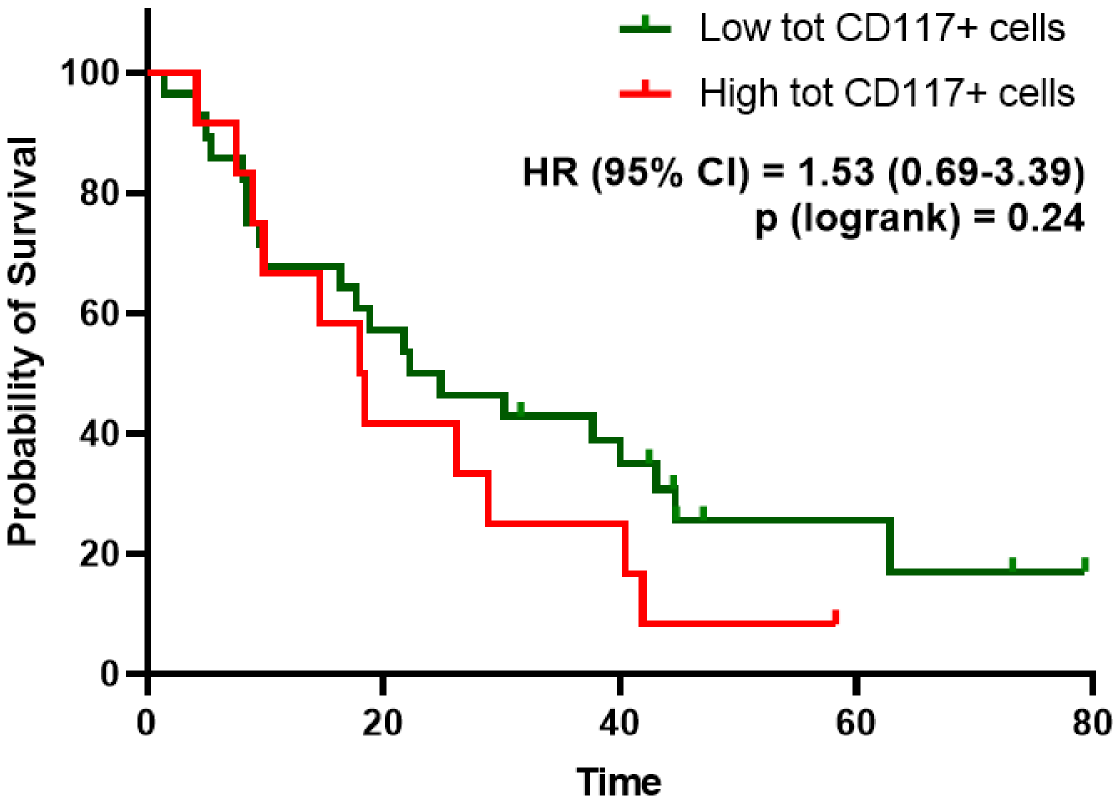

Supplementary Figure 2. Overall survival according to blood levels of CD34+/CD45dim/CD117+ cells. Kaplan–Meier (KM) curves showing the relationship between overall survival and blood concentration of total CD34+/CD45dim/CD117+ cells**.** The logrank test was used for statistical comparisons.
